# Emotion analysis in children through facial emissivity of infrared thermal imaging

**DOI:** 10.1371/journal.pone.0212928

**Published:** 2019-03-20

**Authors:** Christiane Goulart, Carlos Valadão, Denis Delisle-Rodriguez, Eliete Caldeira, Teodiano Bastos

**Affiliations:** 1 Northeast Network of Biotechnology (RENORBIO), Postgraduate Program in Biotechnology, Assistive Technology Group, Federal University of Espirito Santo, Vitoria, Espirito Santo, Brazil; 2 Postgraduate Program in Electrical Engineering, Assistive Technology Group, Federal University of Espirito Santo, Vitoria, Espirito Santo, Brazil; 3 Center of Medical Biophysics, University of Oriente, Santiago de Cuba, Cuba; Boston Children's Hospital / Harvard Medical School, UNITED STATES

## Abstract

Physiological signals may be used as objective markers to identify emotions, which play relevant roles in social and daily life. To measure these signals, the use of contact-free techniques, such as Infrared Thermal Imaging (IRTI), is indispensable to individuals who have sensory sensitivity. The goal of this study is to propose an experimental design to analyze five emotions (disgust, fear, happiness, sadness and surprise) from facial thermal images of typically developing (TD) children aged 7–11 years using emissivity variation, as recorded by IRTI. For the emotion analysis, a dataset considered emotional dimensions (valence and arousal), facial bilateral sides and emotion classification accuracy. The results evidence the efficiency of the experimental design with interesting findings, such as the correlation between the valence and the thermal decrement in nose; disgust and happiness as potent triggers of facial emissivity variations; and significant emissivity variations in nose, cheeks and periorbital regions associated with different emotions. Moreover, facial thermal asymmetry was revealed with a distinct thermal tendency in the cheeks, and classification accuracy reached a mean value greater than 85%. From the results, the emissivity variations were an efficient marker to analyze emotions in facial thermal images, and IRTI was confirmed to be an outstanding technique to study emotions. This study contributes a robust dataset to analyze the emotions of 7-11-year-old TD children, an age range for which there is a gap in the literature.

## Introduction

Studies focused on emotions has increased worldwide, mainly due to their importance for the interpersonal relationship field but also as they are considered potent facilitators of cognitive processes and contributors to many illnesses [1]. In many aspects of daily life, emotions frequently mold social relationships, contributing to communication between humans [2] and enabling the identification of a person’s intention to adopt appropriate responses [3].

In the emotion analysis field, valence (pleasure) and arousal (intensity) are consistent emotion dimensions for emotional perception, which have shown correlation with physiological signals, such as signals from facial muscle activity, skin conductance, heart rate, startle response and brain waves [4–6]. Moreover, efforts for emotion recognition through physiological markers are evident in many studies that show accuracy varying between 60% and 90% using electrocardiography (ECG) [7], electroencephalography (EEG) [8–10] and thermography signals [11–13].

Human faces play an important role in conforming facial expressions and revealing emotions; they are not totally symmetric, with emotions being more strongly expressed in the left side of the face [14]. Facial expressions are derived from both facial muscle activation and influences of the autonomic responses (pallor, blush, pupil size, sweat), revealing threatening or attractive events experienced by the person [15,16]. The set of branches and sub-branches of vessels that innervate the face demonstrates the heating of the skin, which may be related to emotions and studied through Infrared Thermal Imaging (IRTI) [13,17].

IRTI is an upcoming, promising and ecologically valid technique that has been increasingly adopted in studies involving human emotions, which may be associated with physiological parameters [18–20]. In addition, it is a relevant, contact-free technique for people's comfort without the usage of sensors on the body [21].

Although IRTI has been widely employed in studies with adult subjects for emotion analysis and recognition [12,13,22,23], similar studies in children have rarely been addressed [18,24,25]. To the best of our knowledge, an experimental design for emotion analysis by IRTI applied in typically developing (TD) children aged between 7 and 11 years old has not been addressed to date.

The goal of this work is to propose an experimental design to analyze five emotions (disgust, fear, happiness, sadness and surprise) evoked in 7-11-year-old TD children through facial emissivity changes detected by IRTI. An emotion analysis dataset, relative to emotional dimensions (valence and arousal), facial bilateral regions and emotion classification accuracy, was considered.

## Materials and methods

### Participants

This study was approved by the Ethics Committee of the Federal University of Espirito Santo (UFES) (Brazil) under number 1,121,638. Twenty-eight children (12 females and 16 males, age range: 7–11 years old, M = 9.46 years old, SD = 1.04 years old) participated in experiments. Eleven percent were between 7 and 8 years old, and eighty-nine percent were between 9 and 11 years old. The recruited children group was defined taking into account the age range mainly corresponding to middle childhood. The children were recruited through cooperation agreements established with three elementary schools of Vitoria from Brazil. The children’s teachers cooperated with the selection of children according to our inclusion and exclusion criteria; the former consisted of ages between 7 and 11 years old and absence of traumatic experiences and phobias, and the latter consisted of the occurrence of other neurological disorders that affect the development of the brain, usage of glasses and any medicine.

The parents or legal guardians of the children gave written informed consent in accordance with the Declaration of Helsinki. In addition, the children who wanted to participate in the experiments also gave their written terms of assent.

### Recording

For the thermal data acquisition, a Therm-App infrared thermal imaging camera was used, which has spatial resolution of 384 × 288 ppi, temperature sensitivity < 0.07°C, and frame rate of 8.7 Hz. The image normalization was configured to associate lower temperatures with darker pixels (lower emissivity) and higher temperatures with lighter pixels (higher emissivity) with a pixel intensity rate ranging from 0 to 255.

### Stimuli

To evoke emotions in the children, audio-visual stimuli were used, as these are considered the most popular and effective way to elicit emotions [26]. A psychologist supervised the affective video selection, which were obtained from the Internet. Five videos (with duration from 40 to 130 s) were selected to evoke the following five emotions: happiness (funny scenes, compilation of babies laughing), disgust (revolting scenes, such as larvae eaten by humans–beetle larva), fear (tenebrous scenes, with sporadic appearance of haunting–lights out), sadness (compilation of abandoned dogs) and surprise (unexpected scenes, such an animal doing improbable actions, mouse trap survivor–commercial). An additional video with positive emotional content (full movie trailer–Toy Story 3) was also selected to allow better understanding of the experiment.

SAM (Self-Assessment Manikins) [27] was used for affective self-assessment by children for each video; it consists of a point scale from 1 to 9 based on valence (pleasure emotions) and arousal (intensity) dimensions [4,27].

### Procedure

**Fig 1:**
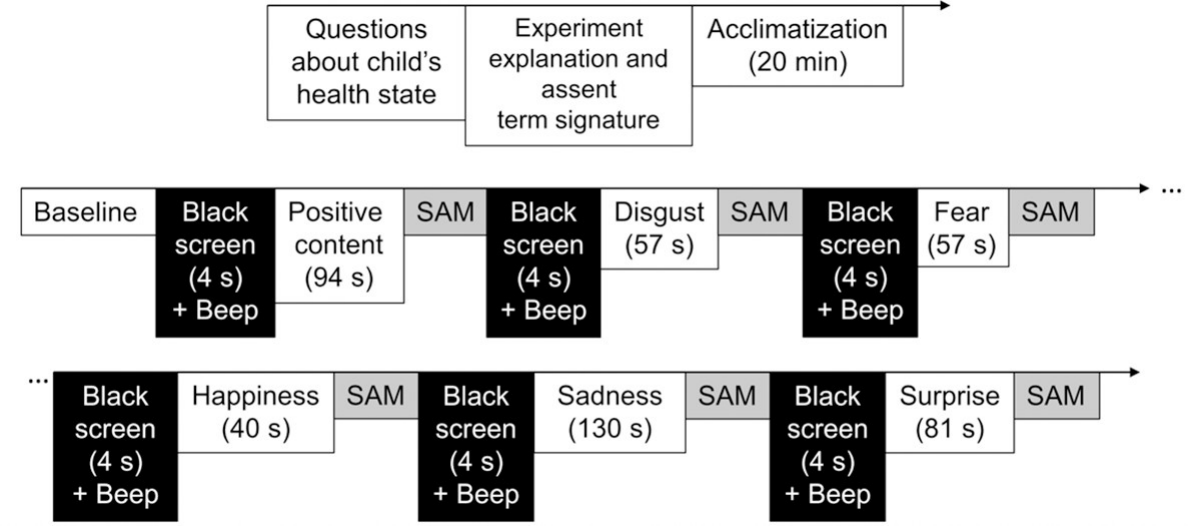
General scheme of the experimental design.

The experiments were carried out in the morning, between 7 a.m. to 12 p.m., with room temperature held at 22°C. The experimental procedure was performed as described in Fig 1.

In the test room, the child was invited to sit comfortably, and questions about her/his health condition were asked (How do you feel today? Any pain in the body? Did you take any medicine these days? Did you practice any physical activity this morning?). These questions were asked because the thermal analysis of the face region may be influenced by some symptoms, such as stuffy or runny nose, sneezing, headache and fever, as well as by physiological changes due to activity of the autonomic nervous system [28,29]. In sequence, the tasks of the experiment were briefly explained, and it was asked if the child really would like to participate. Once the answer was confirmed, the child signed the term of assent after reading it together with the examiner. To avoid and eliminate possible interference during the facial thermal image recording, long hair and fringes were tied and held with a clamp, respectively, and jewelry or diadems were removed. In sequence, SAM, the scale of emotion self-assessment, was explained.

The child was brought into a calm state for at least 20 min in order to adapt her/his body to the room temperature, allowing her/his skin temperature to stabilize for baseline recordings [12,20]. The researcher asked the child not to move and maintain quiet breathing. Meanwhile, brief questions were asked, such as about her/his daily routine, which contributed to the process of relaxation, confidence and proximity to the researcher, with the child becoming more comfortable and less shy or excited during the test.

Next, the child was invited to sit down comfortably on another chair facing a 19-inch screen, with the thermal camera at a distance of approximately 85 cm. The thermal camera was connected to a tablet running Android 4.4.2, in order to acquire the facial thermal images through the Therm-App application.

The child’s head was not kept fixed in a position in order to guarantee the spontaneity of the emotion expression, avoiding any discomfort [29]. However, the child was asked to avoid moving her/his head and putting the hands on the face. In the case of unwanted scenes, she/he was advised to close the eyes, if wished.

The baseline period was recorded before the video display. The affective videos were displayed at the screen to evoke emotions in the following order: positive content video (for training), disgust, fear, happiness, sadness and surprise, according to the psychologist’s guidelines of our research group. To avoid the predominance of negative emotions in the child, the psychologist also recommended that the last video exhibition was positive stimuli, in order to positively sensitize children at the end of the experiment (mechanism called empathy) [30].

Both a black screen (displayed for 4 s) and a beep sound preceded each video, whereas SAM was performed after each one. The child indicated which representation of the SAM corresponded to her/his feeling, and, then, the examiner recorded this information. The experiments were individually performed, and the thermal image recording lasted approximately 11 min in total.

### Thermal data analysis

#### Data pre-processing

**Fig 2:**
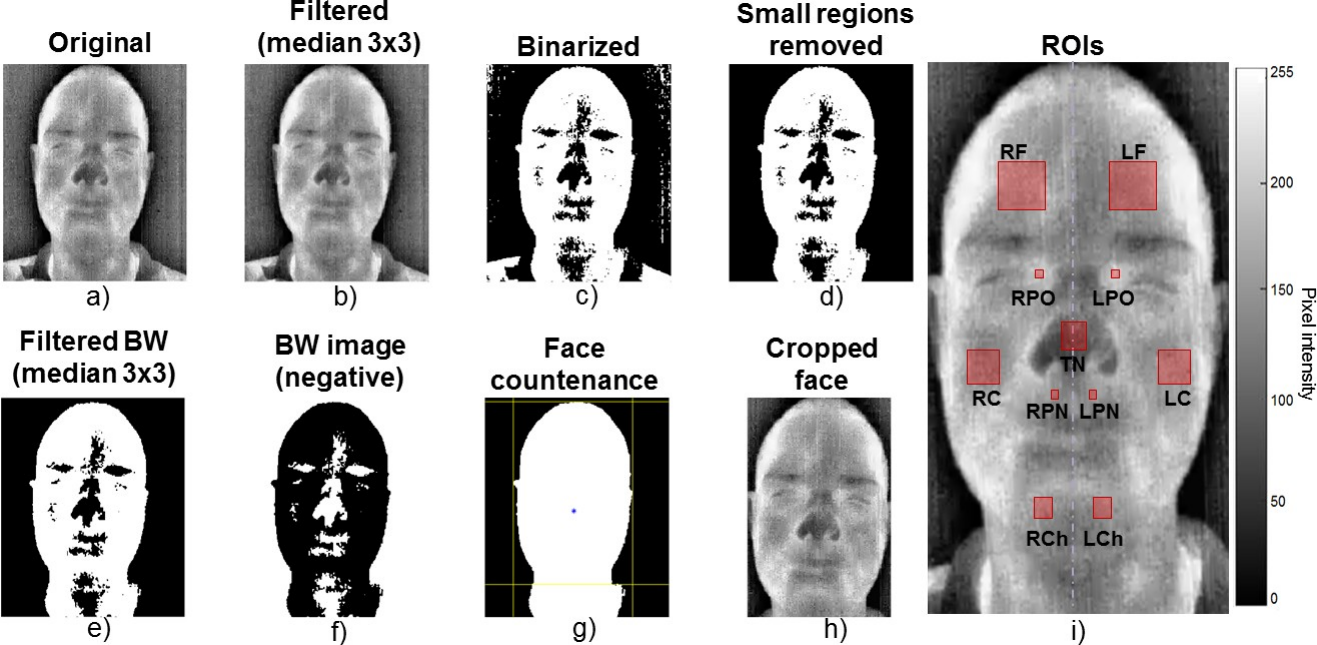
Representation of the pre-processing sequence of the facial thermal images and the eleven regions of interest (ROIs). i) ROIs: LF—forehead (left side); RF—forehead (right side); LPO—left periorbital region; RPO—right periorbital region; TN—tip of nose; LR—left cheek; RC—right cheek; LPN—left perinasal region; RPN—right perinasal region; LCh—chin (left side) and RCh—chin (right side).

Fig 2 shows the pre-processing outcomes for a subject who gave written informed consent to publish his thermal images according to the PLOS consent form.

The images of the faces were extracted from the thermal images using median and Gaussian filters with further binarization to convert the gray scale image into pure black and white (BW). Small sets of non-connected pixels were deleted to improve image quality, facilitating the foreground identification (face—lighter) and background (darker). Using the BW image, it was possible to detect the face boundaries by finding the uppermost point of the head (the uppermost white pixel), and the left and right limits (the leftmost and rightmost pixels, respectively, near the centroid area). Then, a bottom part of the head was inferred by using a proportional distance from the uppermost head point to the centroid. Eventually, this bounding box was applied to the original image, and the region obtained by cropping into the limits of such area was used to further calculate the statistics.

Forehead, tip of nose, cheeks, chin, periorbital and perinasal regions were the facial regions of interest (ROIs) chosen to extract the affective information, as indicated in [20,31]. Squares were manually positioned on the ROIs of the face in the first frame of each set of the thermal images, enabling an automatic square placement on the subsequent frames of the same video. Then, a visual inspection of the ROI bounds was carried out throughout the recordings to ensure the correct position of the squares on the thermal image.

The bounds of each ROI had fixed proportions (width and height) based on the child’s face width [13], such as 6.49% for nose, 14.28% for forehead, 3.24% for periorbital region, 9.74% for cheek, 3.24% for perinasal region, and 5.19% for chin. The ROIs were considered taking into account the bilateral regions of the face in order to analyze the facial thermal symmetry [32,33]. For this purpose, a virtual line symmetrically dividing both sides of the face was used, taking as reference the procerus muscle and the nose. Thus, eleven ROIs were used in our study, as shown in Fig 2I.

#### Feature extraction

Specific segments of the thermal image recordings were considered and selected for the emotion analysis, per child: 3 s of baseline period (before the audio-visual stimuli exhibition, with the face in neutral state and without emotional stimulus) and 30 s corresponding to each affective video (selection from moments with the highest climax of emotional content and elicitation). During the baseline period, the child was sitting comfortably and relaxed, looking to the camera and not moving (see baseline video in S1 Video).

The thermal images were processed by cropping the ROIs in each frame and further calculating their mean, variance and median values (features), as described below.

Let $R^{k}\in R^{m\times n}$ be the ROI described by several pixels *R*_*ij*_ in a range from 0 to 255 (gray scale 8 bits), where *k* is the current ROI being processed and *K* the number of ROIs. It is possible to extract from each ROI the feature vector **F**^*k*^ = {*f*_1_,*f*_2_,…,*f*_14_}, to obtain patterns related to the emotions. The features were extracted from each ROI, as presented in Eq 1.

$$f_{1}=\bar{R}=\frac{1}{m\cdot n}\sum_{i=1}^{m}\sum_{j=1}^{n}R_{ij},$$ (1)

where *f*_1_ is the mean emissivity obtained from all pixels *R*_*ij*_, and *i* and *j* are the rows and columns of **R**, respectively.

$$f_{2}=\sigma^{2}=\frac{1}{(m\cdot n)-1}\sum_{i=1}^{m}\sum_{j=1}^{n}{\left(R_{ij}-\bar{R}\right)}^{2},$$ (2)

where *f*_2_ is the emissivity variance obtained from all pixels.

Similarly, *f*_3_ and *f*_4_ features, which are based on the variance average, were calculated by rows and columns, respectively, as shown in Eqs 3 and 4. Here, ${\bar{R}}_{i}$ is the mean value of the row *i*, while ${\bar{R}}_{j}$ is the mean value of the each column *j*.

$$f_{3}=\frac{1}{m}\sum_{i=1}^{m}\frac{1}{\left(n-1\right)}\sum_{j=1}^{n}{\left(R_{ij}-{\bar{R}}_{i}\right)}^{2},$$ (3)

$$f_{4}=\frac{1}{n}\sum_{j=1}^{n}\frac{1}{\left(m-1\right)}\sum_{i=1}^{m}{\left(R_{ij}-{\bar{R}}_{j}\right)}^{2}.$$ (4)

Additionally, the following features *f*_5_, *f*_6_ and *f*_7_ were computed by the median operator, as shown in Eqs 5 to 7, respectively. *f*_5_ is the median value considering all pixels *R*_*ij*_ in a unique column vector, while *f*_6_ and *f*_7_ were calculated applying the median operator by rows and columns, respectively.

$$f_{5}=median(R),$$ (5)

$$f_{6}=\frac{1}{m}\sum_{i=1}^{m}median(R_{i}),$$ (6)

$$f_{7}=\frac{1}{n}\sum_{j=1}^{n}median(R_{j}).$$ (7)

Finally, the previous seven features *f*_1_ to *f*_7_ were used to compute the other seven features by subtracting similar features of consecutive ROIs, as shown in Eq 8, where *k* is the current ROI being processed.

$$f_{c+7}=\Delta f_{c}(k)=f_{c}(k)-f_{c}(k-1),2\leq k\leq K.$$ (8)

Then, as each frame has 11 ROIs, and each ROI has 14 features, the total number of features was 154.

### Feature selection

For the feature selection, the feature vectors were analyzed according to the training set, searching for those features that minimize the classification errors. Thus, the Neighborhood Component Analysis (NCA) method was used to learn the feature weights using a regularization process [34,35].

Let *T =* {(*x*_*1*_,*y*_*1*_), (*x*_*2*_,*y*_*2*_) …, (*x*_*i*_,*y*_*i*_) …, (*x*_*n*_,*y*_*n*_)} be the training set, where *N* is the number of samples, and *x*_*i*_ is a *m*-dimensional feature vector with class label *y*_*i*_ ∈ {1, 2 …,*C*}. The Mahalanobis distance between the points *x*_*i*_ and *x*_*j*_ is given by Eq 9 [34]:

$$d(x_{i},x_{j})={\left(x_{i}-x_{j}\right)}^{T}W^{T}W(x_{i}-x_{j}),$$ (9)

where *W* is the transformation matrix, and *d* is the Mahalanobis distance. If *W* is a diagonal matrix, Eq 10 can be expressed as follows [35]:

$$d(x_{i},x_{j})=\sum_{l=1}^{d}\omega_{l}^{2}|(x_{il}-x_{jl})|,$$ (10)

where *wl* is a weight associated with the *lth* feature. In particular, each point *x*_*i*_ selects another point *x*_*j*_ as neighbor with probability *p*_*ij*_. Then, a differentiable cost function may be used, which is based on the stochastic (“soft”) neighbor assignment in the transformed space, as shown in Eq 14.

$$p_{ij}=\frac{e^{-d(x_{i},x_{j})}}{\sum_{k\neq i}e^{-d(x_{i},x_{k})}},p_{ii}=0,$$ (11)

$$p_{i}=\sum_{j}y_{ij}p_{ij},$$ (12)

$$\xi (W)=\sum_{i}\sum_{j}y_{i}p_{ij}-\lambda \sum_{l=1}^{d}\omega_{l}^{2},$$ (13)

$$\frac{\partial \xi (W)}{\partial_{\omega_{l}}}=2\left(\frac{1}{\sigma }\sum_{i}(p_{i}\sum_{j\neq i}p_{ij}|x_{il}-x_{jl}|-\sum_{j}y_{ij}p_{ij}|{(x}_{il}-x_{jl})|)-\lambda \right)\omega_{l},$$ (14)

where *p*_*ij*_ is the probability of *x*_*i*_ select *x*_*j*_ as its nearest neighbor; *p*_*i*_ is the probability that the point *x*_*i*_ will be correctly recognized; *y*_*ij*_ = 1 for *y*_*i*_ = *y*_*j*_, and *y*_*ij*_ = 0 otherwise; *λ* is a regularization parameter that can be fitted using cross-validation, and *σ* is the width of the probability distribution. Let *κ* = exp(*z/σ*) be a kernel function with kernel width *σ*. If *σ* → 0, only the nearest neighbor of the query sample can be selected as its reference point, while to *σ* → ∞, all of the points have the same chance of being selected apart from the query point. More details can be found in [35].

### Emotion classification

The five videos used to evoke the five emotions (disgust, fear, happiness, sadness and surprise) were labeled. For this purpose, segments of 30 s from each video were labeled as having a high potential to trigger the desired emotion. Thus, groups of patterns linked to a same emotion were obtained.

To evaluate the emotion recognition, the training and validation sets were chosen for several runs of cross-validation (*k* = 3). Here, both training and validation sets were formed in each run, selecting only patterns that correspond to the same segment. Afterwards, on each set, 11 ROIs were located on the face to obtain feature vectors (154 features) (see details in Feature extraction section).

The feature vectors of the training set were analyzed through a supervised method for feature selection (of low computational cost) based on NCA [34,35]. Thus, the dimensionality of these feature vectors was reduced, enhancing the class separation and the classification stage. In this instance, the feature vectors of the training set were normalized, using both mean and standard deviation values as reference. Then, the validation set was reduced, taking into account the relevant features, and normalized using the same reference values (mean and standard deviation) obtained from the training set. Finally, Linear Discriminant Analysis (LDA) was used as classifier [36,37] (see dataset for emotion recognition in S1 Dataset).

### Statistical analysis

Means and standard deviations were calculated to evaluate the valence and arousal dimensions from SAM. Average values of the emissivity variations were calculated to obtain the mean heat signature in each ROI throughout emotion elicitation, taking as reference the baseline period. Thus, emotion data from the affective videos were compared with data from the baseline. A comparison between the bilateral ROI data of the face was also accomplished to verify the facial thermal asymmetry related to the evoked emotions. Student’s *t* test (*α* = 0.05) using a Bonferroni correction was used to verify the significance of such emissivity variations in the facial ROIs. In this study, the data normal distribution was verified applying the Kolmogorov-Smirnov test, rejecting the null hypothesis (normal distribution) at the 5% significance level. For this reason, a logarithmic transformation with base two was applied on the data before applying Student’s *t* test. Furthermore, indices such as accuracy, true positive rate (TPR), Kappa and false positive rate (FPR) were used to evaluate the emotion classification.

## Results

### Valence and arousal analysis

The mean SAM scores for valence and arousal dimensions calculated for the 28 participant children are shown in Table 1.

**Table 1:** Means and standard deviations (SD) of SAM performed by 28 children.

Disgust		Fear		Happiness		Sadness		Surprise	
Valence Mean (SD)	Arousal Mean (SD)	Valence Mean (SD)	Arousal Mean (SD)	Valence Mean (SD)	Arousal Mean (SD)	Valence Mean (SD)	Arousal Mean (SD)	Valence Mean (SD)	Arousal Mean (SD)
2.64 (1.64)	4.93 (2.69)	2.96 (1.69)	5.71 (3.28)	8.68 (0.82)	5.36 (3.03)	1.68 (1.44)	3.25 (2.69)	7.93 (1.49)	5.68 (3.29)

From Table 1, it is possible to see that the valence scores were pronounced (close to the extreme values of the scale, 1 and 9, corresponding to negative and positive emotions, respectively), whereas arousal scores, close to number five, exhibited a general moderate intensity. Although arousal scores were not substantial, valence scores obtained suggested that the affective videos did trigger specific emotions in TD children.

### Thermal data analysis

#### Feature selection

**Fig 3:**
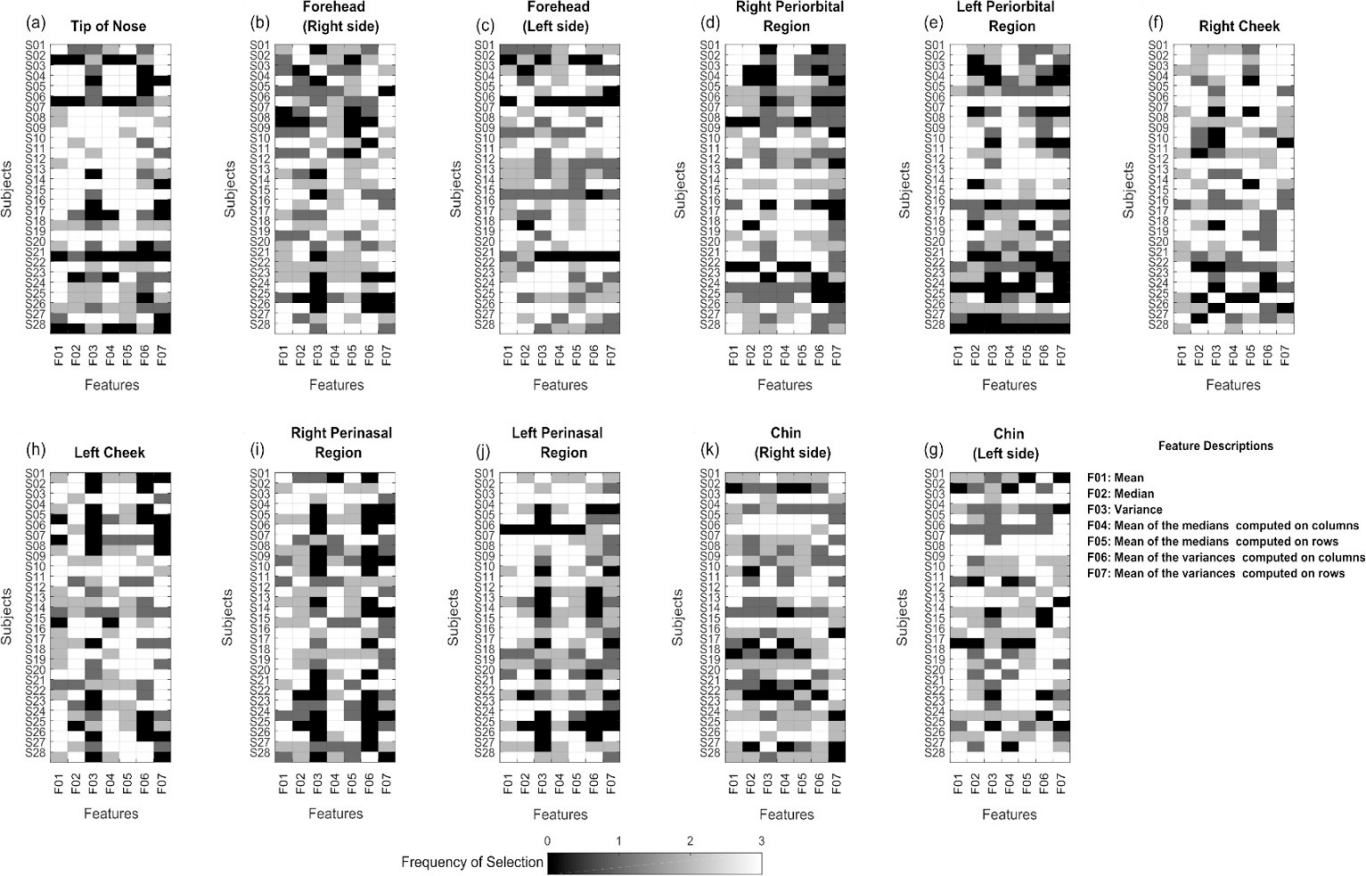
Feature selection maps for each ROI.

Fig 3 shows the feature selection frequency from each ROI for each child, considering three cross-validations for the random selection of the affective segments. The tip of the nose was highlighted due to its high feature selection frequency, shown by white squares in Fig 3A.

**Fig 4:**
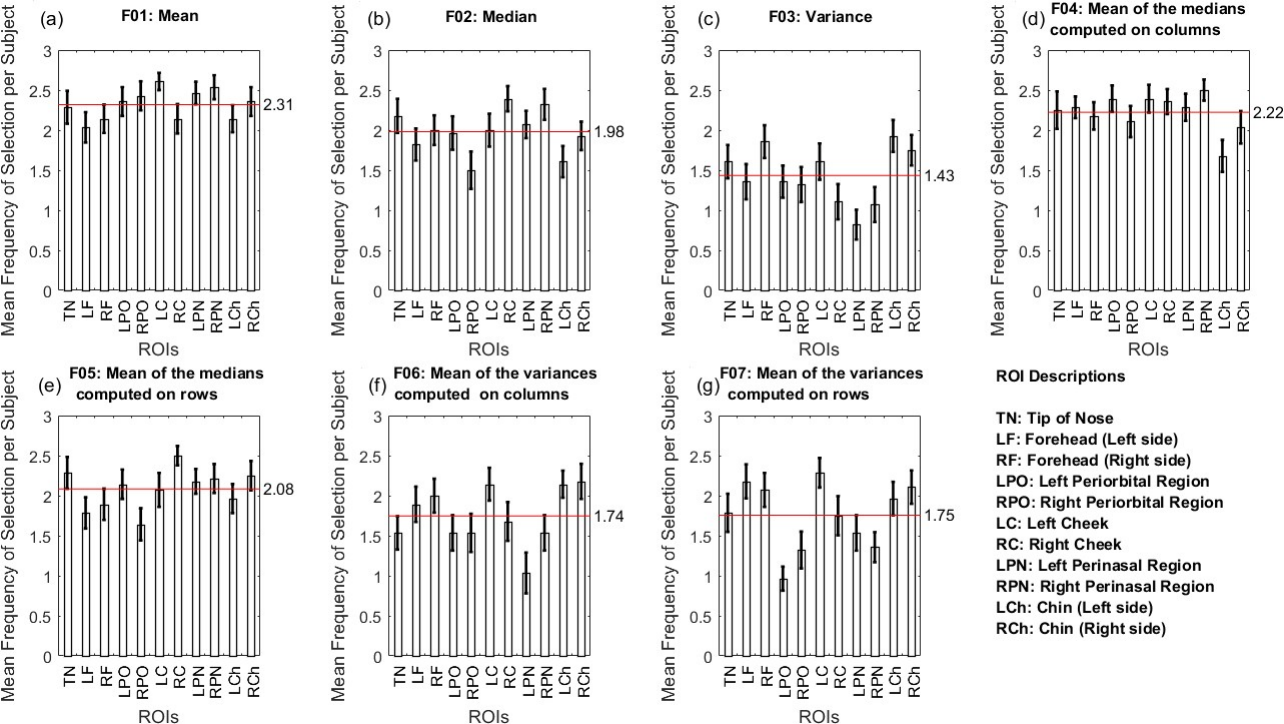
Mean frequency of feature selection per ROIs per subject.

Fig 4 shows the contribution of each ROI in the feature selection frequency. Seven features were selected: mean, median, variance, mean of the medians on columns, mean of the medians on rows, mean of the variances on columns and mean of the variances on rows. The highest mean values of selection frequency were mean (2.31) and mean of the medians (2.22 and 2.08, for columns and rows, respectively).

Both Figs 3 and 4 show the independent contribution of the bilateral ROIs for the feature selection, inferring the facial thermal asymmetry.

#### Emotion classification

**Fig 5:**
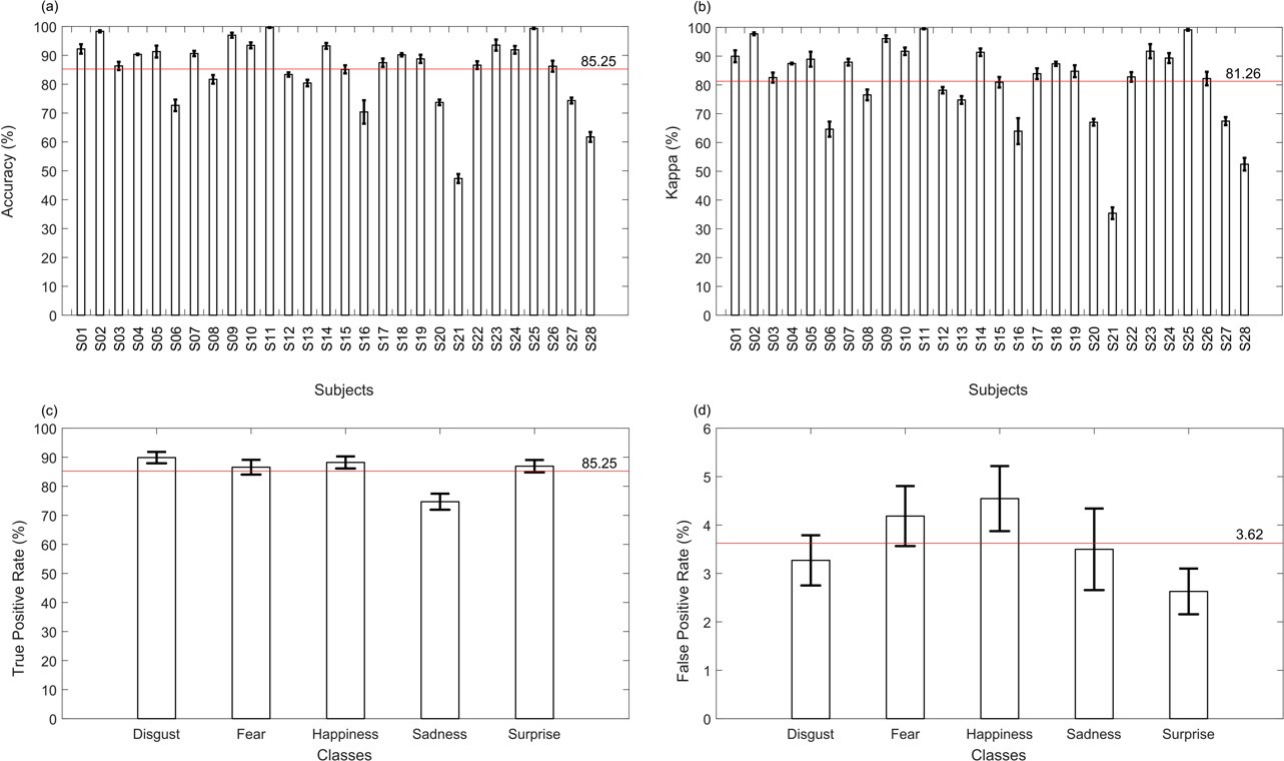
Performance of the emotion classification.

The results obtained by the classification for the five emotions had a mean accuracy higher than 85% for the 28 subjects (Fig 5A), with Kappa higher than 81% (Fig 5B). Moreover, the true positive rate was higher than 80% for four emotions, except sadness (Fig 5C). The accuracy reached by the classifier was of 89.88% for disgust, 86.57% for fear, 88.22% for happiness, 74.70% for sadness, and 86.93% for surprise. On the other hand, the false positive rate had a mean value of 3.62% and values lower than 5% for classification errors, with 3.27% for disgust, 4.18% for fear, 4.54% for happiness, 3.50% for sadness and 2.63% for surprise.

A database and an algorithm for emotion classification can be viewed in S1 Dataset.

#### Facial emissivity variation

**Fig 6:**
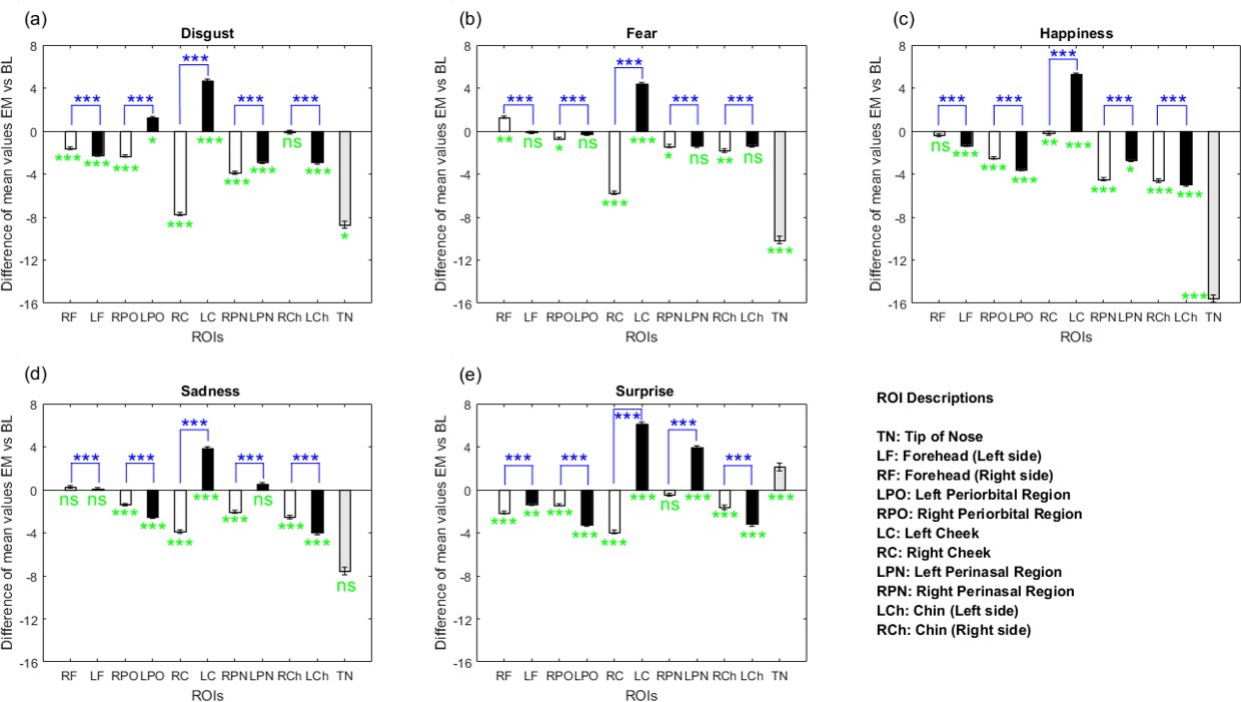
Emissivity variation analysis in the selected ROIs, considering the five emotions, taking as reference the baseline (in green) and the ROI pairs (in blue). The green highlights (below line 0) indicate the significance of emissivity variations in the ROIs triggered by each emotion in relation to the baseline. The blue highlights (above line 0) indicate the significance of emissivity variations occurred between the bilateral ROIs to verify the facial thermal asymmetry. Legend: EM vs BL means Emotion versus Baseline; ns means no significant emissivity variation (*p-value* > 0.05), while * (*p-value* ≤ 0.05), ** (*p-value* ≤ 0.01) and *** (*p-value* ≤ 0.001) indicate significant emissivity variation.

**Fig 7:**
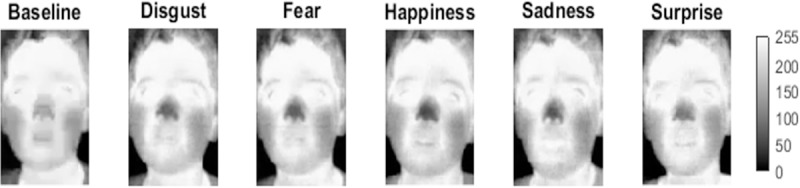
Representative frames of the emissivity decrease (in relation to baseline) in nose of a child during the emotions. Pixel intensity: 0–255.

For the emotion analysis in comparison to the baseline period, the mean emissivity variations were calculated from the eleven ROIs (LF: forehead (left side); RF: forehead (right side); LPO: left periorbital region; RPO: right periorbital region; TN: tip of nose; LR: left cheek; RC: right cheek; LPN: left perinasal region; RPN: right perinasal region; LCh: chin (left side) and RCh: chin (right side)). The pixel values from all ROIs were used for each analyzed frame. Fig 6 shows significant emissivity variations generated by the emotions in the ROIs in relation to the baseline period, considering the thermal tendency (increasing, decreasing or stable). Moreover, Fig 6 shows significant emissivity variations generated by the emotions between the bilateral ROI pairs. Fig 7 shows emissivity decrease (in relation to baseline) in the nose of a child during the emotions. The child’s parents gave written informed consent to publish his thermal images according to the PLOS consent form.

As a result, significant emissivity decreases were observed in the tip of the nose for disgust, fear and happiness, periorbital regions for happiness, sadness and surprise, perinasal regions for disgust and happiness, chin for happiness, sadness and surprise, and forehead for disgust and surprise (Fig 6).

Fig 6 shows significant emissivity variations between all ROI pairs for the five emotions, evidencing the thermal asymmetry of the face. In ROI asymmetry analysis, it is possible to notice the significant variations between the cheek pairs with divergent thermal tendencies (emissivity increases in the left cheek and decreases in the right one) for the five emotions. Moreover, divergent thermal tendencies were observed between periorbital region pairs for disgust and perinasal region pairs for sadness and surprise. Fig 6 also shows a significant variation in the right periorbital region and right cheek, with thermal decreases, and left cheek, with thermal increase, for all emotions.

## Discussion

### Feature selection and emotion classification

According to Figs 3 and 4, tip of nose, cheeks (especially the right one) and chin (mainly its left side) presented higher contribution of features over all cross-validation. This way, the features’ means and mean of medians were highly selected, as shown in Fig 4, with a similar contribution of ROIs located on the right side of the cheeks, left periorbital and perinasal regions.

A growing number of studies aim at an automatic classification of emotions through extraction of information from thermal images [11–13]. The classification method based on LDA used here to recognize five emotions (disgust, fear, happiness, sadness and surprise) reached mean accuracy of 85.25% and Kappa of 81.26%. Specifically, accuracy values of 89.88% for disgust, 88.22% for happiness, 86.93% for surprise, 86.57% for fear and 74.70% for sadness were achieved, confirming the effectiveness of our proposed experimental design.

Regarding the emotion identification and classification through IRTI of other works with adults, our classification performance was similar. For example, in [13], the authors proposed a system that achieved accuracy of 89.90% using another emotion set (anger, happiness, sadness, disgust and fear) with twenty-five adult subjects. In [12], the authors proposed to distinguish between baseline and affective states of twelve adult subjects in (high and low) levels of arousal and valence through visual stimuli (static images). These researchers found an accuracy of approximately 80% between baseline and high arousal and valence levels, while for baseline versus low arousal and valence levels, they obtained accuracy of 75%. In [38], a deep Boltzmann machine (DBM) was used for emotion recognition from thermal infrared facial images through thermal databases of facial expressions, considering 38 adult subjects and evaluating the valence recognition (positive versus negative). They obtained accuracy of 62.9% for classification of negative and positive valence. In [22], a computational model of facial expression recognition of face thermal images was proposed, using eigenfaces to extract features from a face image dataset of only one adult subject, through Principal Component Analysis (PCA). The evaluated emotions were anger, happiness, disgust, sad and neutral, and the proposed system reached accuracy of approximately 97%. In [39], histogram feature extraction and a multiclass Support Vector Machine (SVM) were used as emotional analysis method to classify four emotions (happiness, sadness, anger and fear) from thermal images of 22 subjects available in the Kotani Thermal Facial Expression (KTFE) Database. The authors achieved a classification average accuracy of 81.95%. In [40], the authors used thermal image processing, Neural Network (NN) and Back Propagation (BP) to recognize neutral, happy, surprise and sad facial expressions of one female, obtaining a mean accuracy of 90%.

### Valence and arousal analysis and ROIs

The valence dimension represents the state between unhappiness and happiness, whereas arousal is the state between relaxation and activation [41]. In our study, the valence values were pronounced towards the extremities (1 and 9) of the SAM rating (Table 1). Fig 6 showed that happiness, surprise and disgust were the inducers of the greatest emissivity variations in the children’s faces, in relation to the baseline. Such emotions triggered significant emissivity variations in pairs of the periorbital region and cheeks and nose. In general, the ROIs that had more significant emissivity variations were the cheeks, periorbital regions and nose.

Some studies highlighted the association between thermal variation in the face and emotional dimensions (valence and arousal), such as in [32]. The authors reported higher correlation between facial thermal changes and arousal than valence, with stimuli obtained from a picture database. In fact, many findings on thermal variations note temperature changes for high arousal settings, mainly associated with high anxiety levels [17,25,42].

In literature, for valence dimension, temperature increments in brows, cheeks and around the eyes were observed in adults, with brows and cheeks related to negative valence and eyes related to positive valence [16]. On the other hand, temperature decrements in forehead, cheek and nose were evidenced in babies, along with pleasant emotions [24]. The work described in [43] evaluates the thermal variation in the perinasal region, considering facial expressions, in order to distinguish examples of negative (unpleasant) and positive (pleasant) arousal, distress and eustress conditions, respectively. For eustress (with positive facial expressions), they found locally elevated perinasal signal, whereas for distress (with negative facial expressions), they found fluctuating perinasal signal. The observed differences can be related to the muscle deformation presented during the facial expression, beyond perspiration (found in distress conditions). Therefore, valence effects could be related to muscular deformations due to facial expressions.

In general, for valence, decreased facial temperature is considered a sign of negative emotions [24], which is confirmed in Fig 6. This predominant decrease observed in most ROIs may be either the reflex of the subcutaneous vasoconstriction under the control of a sympathetic activation mediating the central activation [15,20] or perspiration, a physiological phenomenon from the sympathetic autonomous system, which occurs due to the absorption of the latent heat by perspiration pore activation, decreasing the local thermal emission [44]. Such decreases were detected in the ROI pair of the forehead region during disgust and surprise; the periorbital region during happiness, sadness and surprise; the perinasal region during disgust and happiness; the chin during surprise, happiness and sadness; and finally, the nose during disgust, fear and happiness. On the other hand, forehead was the ROI that had the least variation of emissivity compared to other ROIs. The tendency towards no significant changes in the forehead is consistent with the fact that it has the most stable temperature [20,32]. In general, emissivity decreases were mainly found in the right cheek and right side of the periorbital and perinasal regions for negative emotions (disgust, fear and sadness), and in the periorbital region and chin for positive emotions (happiness and surprise). Decreases in the right cheek and increases in the left cheek were observed for all emotions.

For negative (disgust and fear) and positive (happiness) emotions, a significant emissivity decrement in the nose was particularly noticed (see Fig 6 and Fig 7), in accordance with findings in the literature that indicate nasal temperature decreases during stress, startle and happiness situations [13,15,20]. Thus, it is possible to suppose the contribution of the valence dimension to the nasal thermal decrement. On the other hand, an emissivity increase occurred in the nose for surprise, such as shown in Fig 6. During the surprise video, a fright moment occurred, which may have caused a momentary increment in the heart rate, generating a temperature increase in the face and triggering a vasodilation in the region of the nose [31].

The nose is the most consistent indicator of stress and negative emotions [15,31], presenting solid results, as it is not considered to be greatly affected by expressions. In fact, it is affected by sympathetic responses, such as subcutaneous vasoconstriction or perspiration, which trigger thermal decrements [15,20,44]. Decrease in nasal temperature was observed in distress signs during the investigation of guilt in children with ages between 39 and 42 months [18], and in mild posttraumatic stress disorder subjects exposed to a sudden acoustic stimulus in a fear conditioning perspective [19]. Moreover, many authors consider the nasal temperature as an indicator of affective states in animals [20]. For example, in fear contexts, temperature drops were detected in the noses of monkeys submitted to settings with a threatening person [45], as well as in monkeys exposed to negative audiovisual, with only audio or visual stimuli [46].

Fig 3 reveals the high contribution of the nose in relation to other ROIs for the feature selection frequency. In fact, nose (tip of nose) is an important and much-studied marker for investigation of thermal variations in the face of humans and animals, specifically in distress and fear contexts [15,18,19,45,46].

### Bilateral facial thermal variation

The facial asymmetry is easily revealed in the emotional expressions [14], and a thermal difference greater than 0.5°C might indicate clinical thermal asymmetry [33]. Fig 6 demonstrated significant emissivity differences between all the ROI pairs, inferring thermal asymmetry triggered by emotions. Moreover, a thermal tendency difference in cheeks was observed, with a general thermal decrease on the right side and an increase on the left one. Although there was a significant decreasing thermal tendency for most ROIs, significant emissivity increases were presented in the left cheek and perinasal region for surprise, and in the left cheek and periorbital region for disgust. Moreover, Fig 4 showed the relevance of some bilateral ROIs for the feature selection, presented by the cheek pair, for example.

The asymmetry of the face might be related to the brain lateralization, in which the right hemisphere plays a dominant role in emotion processing [14,47,48]. There is a dominance of the facial left side for emotion expressions, due to its innervation by the right hemisphere, which is dominant for facial emotional expression [49]

## Study limitations

The focus of this work was the emotion analysis by IRTI elicited in typical children of developmental ages, for which there is a gap in the literature. Our method presents some limitations, and one is related to the absence of an automated ROI tracking and positioning method. In our work, manual ROI positioning was performed in the first frame of each thermal video, which was used as a reference for the next ROI marking, being automatically propagated to the rest of the video. This was made to ensure the correct positioning of the ROIs. The advantage of using a manual check method is to discard ROIs that are not positioned correctly, assuring an appropriate selection of frames for our analyses. Automatic positioning of ROIs and their tracking methods are subjects for future works, as real-time face tracking could reduce the frame discarding due to ROI positional error or head movements. It could also ensure a correct ROI positioning even during such movements. An example of a facial tracking method could be the combination of particle filtering with a probabilistic template algorithm (spatiotemporal smoothing template) proposed in [50], which enables a fast, flexible and accurate tracker in spite of head movements and physiological changes.

Another limitation of our work is the possible presence of combinations of ROI positional and muscle deformation errors due to facial expressions, which may have produced some of the effects found in our results, as such emissivity decrease in the right cheek and increase in the left one. This facial expression variability due to muscle deformation can affect the thermal variations, and a potential investigation could associate automated methods of both thermal and visual imagery analysis for the recognition of such expressions, as cited in [51], as the fusion of both methods allows a better accuracy compared to the individual modalities.

The fixed size rectangles of the ROIs are also considerable limitations. For example, the size of the periorbital ROI is tiny, and even a few pixels of motion error could affect the statistics. In addition, given that the face covers the entire frame, even a small face motion in actual space may translate to a potential shift in the image plane.

Another limitation is related to the recruited children’s developmental ages, between 7 and 11 years old. This age range is large from the developmental point of view; a 7-year-old child is very different from an 11-year-old child in terms of emotional maturity, for instance. In future analyses, a tighter age range (7–8 or 10–11 years old, for example) could be considered. In fact, neuroscience studies have suggested that social and emotional learning is best prior to 6 years of age (early childhood), contributing to future emotional competence and emotion knowledge of children [30]. Despite developmental differences, our sample of children was defined taking into account the age range mainly corresponding to middle childhood.

Finally, the experimental sessions of our study were not counterbalanced, which also characterizes a limitation. Future studies should display the emotional videos in another order to verify the best sequence of stimuli to generate a greater differentiation between the emotions. In spite of this limitation, the psychologist of our research group approved the display order addressed in our work, which was performed for all children in the experiments.

## Final considerations

The proposed experimental design was carried out in a careful way with typically developing children aged between 7 and 11 years, in which five emotions (disgust, fear, happiness, sadness and surprise) were evoked by audio-visual stimuli, triggering significant facial emissivity variations in 11 facial ROIs, as recorded using IRTI. An analysis set of emotions considered emotional dimensions, facial bilateral ROIs and emotion classification. To the best of our knowledge, to date, there is no similar experimental design applied in children of this age group in literature.

The emissivity variation analysis through IRTI was demonstrated to be efficient, indicating significant variations in facial ROIs. This can note the ROIs and emotions that deserve attention in future studies with children, which may be more assertive in identifying quantitative patterns for emotion recognition. In addition, IRTI showed to be a valuable touchless technique for emotion analysis.

Our main findings reflect the efficiency of the proposed experimental design and become part of an important data set for support in future work. The main findings were the following: a) mean and means of medians (on columns and rows) were the features with the highest average values for the selection frequency; b) tip of nose, cheeks and chin were the most contributing ROIs for the feature selection; c) a high accuracy (higher than 85%) was obtained for the classification of the five emotions; d) SAM showed valence with pronounced values, with a predominant thermal decrease observed in almost all ROIs in relation to the baseline; e) disgust, happiness and surprise induced the greatest significant emissivity variations in the children’s face; f) thermal decrease in the tip of the nose indicated its relevance as a potential marker for affective states, including for valence; g) cheeks presented significant variations for all emotions; and h) a facial thermal asymmetry was evidenced through distinct significant variations between all the bilateral pairs, with predominant thermal trend differences in cheeks.

Further validation of the results in a larger sample of children is necessary, which is challenging, due to their spontaneous behavior. Therefore, appropriate planning and careful execution of the experimental design is important, in order to extract the most believable information from children.

The experimental design carried out here provided interesting and promising results, generating a robust dataset that can be useful for future studies about emotions and behaviors of children.

## Supporting information

S1 Video Baseline video. Short video corresponding to the baseline period of a child with positioned ROIs.
(AVI)

S1 Dataset Dataset for emotion recognition. Code and database used for emotion classification.
(ZIP)
